# Diagnosis of Lisinopril-Induced Small Bowel Angioedema After a Seven-Year Diagnostic Journey: A Case Report With Patient Perspective

**DOI:** 10.7759/cureus.108019

**Published:** 2026-04-30

**Authors:** Stephanie Sutton, Joyce Paulson

**Affiliations:** 1 College of Medicine, University of Central Florida, Orlando, USA; 2 Department of Internal Medicine, University of Central Florida, Orlando, USA

**Keywords:** ace-i, ace inhibitor angioedema, adverse drug reaction, bradykinin mediated angioedema, complication of treatment, lisinopril adverse effects, recurrent abdominal pain, small bowel angioedema, target sign, visceral angioedema

## Abstract

Angiotensin-converting enzyme inhibitors (ACEIs) are widely prescribed for managing hypertension and improving cardiovascular and renal health while being relatively low-cost and generally well-tolerated. ACEI-induced angioedema is an uncommon but potentially serious adverse effect that may present with swelling of the face, lips, tongue, and pharynx, raising concern for possible airway obstruction, hypoxia, and even death. Other presentations have been documented, including small bowel angioedema (SBAE). SBAE is diagnostically challenging due to its nonspecific gastrointestinal symptoms. This report highlights a middle-aged woman who experienced ACEI-induced SBAE for nearly seven years alongside comorbid conditions. Despite extensive evaluations for gastrointestinal pathologies, her condition remained unresolved until a multidisciplinary team attributed her symptoms to ACEI therapy. Upon discontinuing lisinopril, her symptoms resolved, emphasizing the importance of recognizing drug-induced causes in patients with unexplained, persistent symptoms. This case underscores the critical role of the healthcare team in identifying and managing adverse drug reactions (ADRs), especially with common medications like ACEIs. Clinicians may utilize readily available assessment tools to help identify possible ADRs. A multidisciplinary approach is essential, as physicians across specialties are uniquely positioned to recognize potential ADRs and communicate concerns collaboratively. Early detection can reduce unnecessary hospitalizations and interventions and improve patient outcomes.

## Introduction

Angiotensin-converting enzyme inhibitors (ACEIs) are among the most frequently prescribed drugs in the United States, with nearly 40 million people estimated to be receiving therapy [1]. These drugs are widely used for their effectiveness in managing hypertension and optimizing cardiovascular and renal health while being relatively low-cost and safe. However, angioedema, a rare but serious side effect, occurs in 0.1-0.7% of patients, as determined by several multicenter studies across multiple countries [1,2]. Presentation typically occurs within the first few months of starting treatment, but can also arise after years of ongoing therapy.

The incidence of ACEI-induced angioedema is higher in certain populations, including women, older individuals, Black Americans, smokers, and those with a history of drug rash, seasonal allergies, or immunosuppressive therapy [1,3]. For women, estrogen exposure is believed to be a specific risk factor, as estrogen induces prekallikrein or the bradykinin type 2 (B2) receptor [1,3,4]. For Black patients, the exact cause is unknown; however, gene polymorphisms have been identified that influence bradykinin production [3,5].

Angioedema often presents visibly, with swelling of the face, lips, periorbital, and oropharyngeal regions, raising concern for possible airway obstruction, hypoxia, and even death [1]. Less common presentations of angioedema have also been documented, such as small bowel angioedema (SBAE) [6]. These cases often pose diagnostic challenges due to their nonspecific symptoms and overlap with other gastrointestinal complaints.

This report focuses on the diagnostic challenge of a woman who was affected by ACEI-induced SBAE for nearly seven years. Her case was further complicated by a personal and family history of gastrointestinal pathologies, underscoring the need for heightened awareness and vigilance. This report also explores the role of clinicians in recognizing and addressing this rare yet significant phenomenon.

## Case presentation

Our patient is a middle-aged woman who presented to the emergency department (ED) in March 2015 with the sudden onset of abdominal pain. Her medical history was notable for postmenopausal symptoms, rosacea, and essential hypertension, which had been managed with lisinopril since August 2014, less than one year prior to presentation. Her antihypertensive regimen was adjusted over time, from lisinopril 10 mg to 20 mg daily, followed by transition to hydrochlorothiazide-lisinopril 12.5 mg/20 mg and subsequent reduction to 12.5 mg/10 mg. The patient did not initially indicate a family history pertinent to her presentation; however, her family history is notable for cervical and liver cancer, thyroid disease, hyperlipidemia, anemia, hypertension, and heart disease in immediate family members.

During her initial visit, the abdominal pain was localized to the right upper quadrant (RUQ), which radiated to the epigastric region. She described the pain as cramping in nature, moderate to severe in intensity, and accompanied by nausea and vomiting. She denied systemic symptoms such as fever, chills, night sweats, or unintentional weight loss. After an initial evaluation that ruled out acute abdominal emergencies, the patient was discharged home with symptomatic management. However, she returned to the ED two weeks later with the same symptoms and again two weeks after that, indicating a recurrent pattern.

Over the next two years, the patient continued to follow up with her primary care physician at a University of Central Florida-affiliated clinic and consulted several gastroenterologists, resulting in a significant workup for possible pathologies. Despite these efforts, no definitive cause for her persistent abdominal pain was identified, and her pain remained severe enough, prompting numerous visits to the ED. Between July and November of 2017 alone, she visited the ED five times.

**Figure 1:**
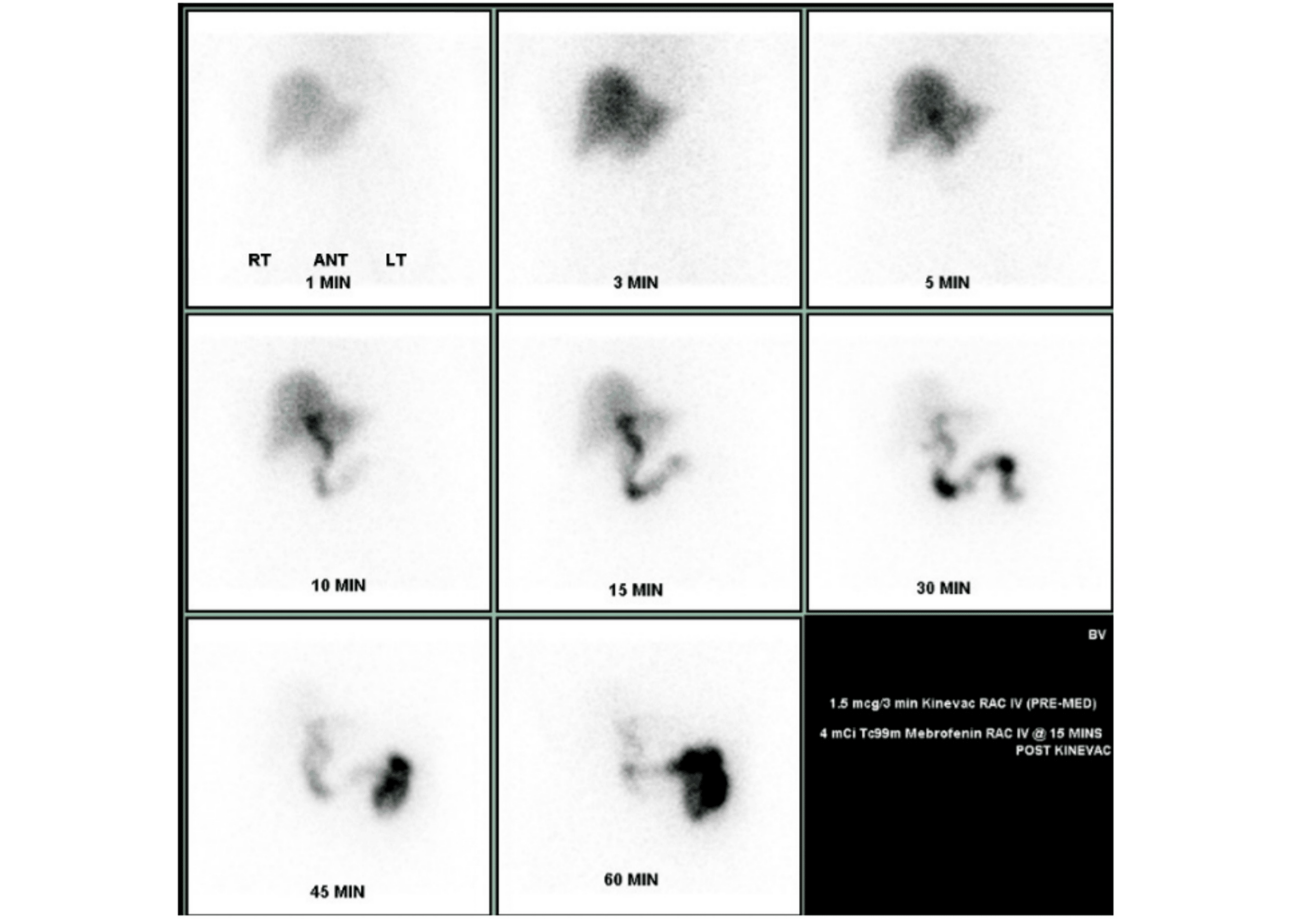
HIDA (hepatobiliary iminodiacetic acid) scan demonstrating normal biliary kinetics, with a total score of 2 (normal <6), consistent with a normal sphincter of Oddi study

In October 2017, an endoscopic ultrasound (EUS) identified biliary sludge, suggesting a potential source for her symptoms, despite prior normal biliary imaging and function on a hepatobiliary iminodiacetic acid (HIDA) scan and labs. See Figure 1 for the HIDA scan. EUS imaging is unavailable.

The patient also revealed an extensive family history of cholecystectomies in female relatives. During a preoperative appointment in November 2017, the patient reported severe abdominal pain, leading to hospitalization and an expedited cholecystectomy. For five months following the surgery, the patient had no complaints of abdominal pain over the course of four routine visits with her physicians. This period of quiescence supported the hypothesis that her pain had been biliary in origin.

However, by May of 2018, the patient’s pain returned, accompanied by frequent visits to physicians and the ED. Additionally, a family member had received a diagnosis of Crohn's disease. Complement testing was conducted, showing normal C1 esterase inhibitor functional (100%) and C1 esterase inhibitor protein (31 mg/dL) levels, as well as a normal C4 level (17 mg/dL). These findings help exclude hereditary or acquired C1-inhibitor deficiency, though they do not confirm ACEI-induced angioedema. Immunoglobulin levels were also within normal limits for IgM (85 mg/dL), IgA (218 mg/dL), and IgG (960 mg/dL) (Table 1).

**Table 1:** Complement and immunoglobulin laboratory evaluation

Laboratory Test	Patient Value	Reference Range
C1 Esterase Inhibitor, Functional	>100	≥68%
C1 Esterase Inhibitor, Protein	31	21–39 mg/dL
C4	17	15–57 mg/dL
IgM	85	48–271 mg/dL
IgA	218	81–463 mg/dL
IgG	960	694–1618 mg/dL

By 2019, her symptoms escalated to include hyperemesis and diarrhea, with bowel movements sometimes occurring up to ten times per day. She was started on budesonide and transitioned to a strict gluten-free diet for presumed inflammatory bowel disease and celiac disease, respectively. Concern for infectious etiology led to the prescription of antibiotics on several occasions. Further, during this period, magnetic resonance enterography (MRE) incidentally identified a cystic lesion with non-suspicious features on her pancreatic tail.

**Figure 2:**
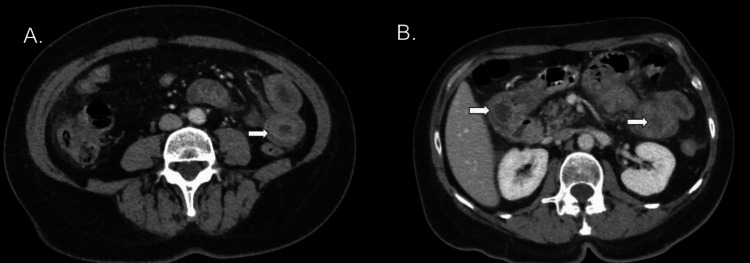
Axial contrast-enhanced CT abdomen and pelvis demonstrating findings consistent with ACEI-induced small bowel angioedema (A) August 2020; (B) December 2022. White arrows highlight the characteristic "target sign" and segmental bowel wall thickening. These images are original diagnostic CT scans from the patient described in this report; informed consent for publication was obtained.

Despite interventions, clinic notes in 2021 reported persistent, severe pain, although the patient expressed reluctance to return to the ED. Extensive testing continued throughout 2022, but yielded no further diagnostic leads until December 2022. The patient had another CT, and the report stated, "Small bowel enteritis involving loops of bowel within the left upper and lower quadrant. Note, the location is nearly identical to the prior CT scan from 2020. If the patient is on an ACE inhibitor, angioedema would be a concern” (Figures 2A, 2B).

At a follow-up appointment in March 2023, a gastroenterologist reaffirmed that lisinopril, prescribed for hypertension since 2014, might be the underlying cause of her symptoms. Collaborating with her cardiologist, a decision was made to hold her lisinopril. She subsequently transitioned to hydrochlorothiazide (HCTZ) alone, and symptoms resolved.

Since discontinuing lisinopril, the patient has had no abdominal pain or ED visits and denied symptoms at both her September 2023 gastroenterology follow-up and annual exam. In early 2024, she resumed eating gluten without adverse effects, following years on a restrictive gluten-free diet due to concern for possible celiac disease. By September 2025 (30 months post-ACEI discontinuation), she reported increased energy and exercise tolerance with continued absence of pain. Blood pressure remains controlled on HCTZ alone, and GERD symptoms are mild and well managed with famotidine and pantoprazole without affecting quality of life.

Additionally, the patient has included her perspective in a supplemental letter.

## Discussion

The pathophysiology of ACEI-induced SBAE is primarily attributed to inhibition of angiotensin-converting enzyme (ACE), which normally degrades bradykinin and substance P [3]. Inhibition of ACE leads to accumulation of the vasoactive nonapeptide bradykinin, which binds constitutively expressed kinin B2 receptors on endothelial cells, promoting nitric oxide release, vasodilation, and increased vascular permeability [7]. Consequently, ACEI-associated angioedema is classified as a form of bradykinin-mediated angioedema. Impaired degradation of substance P further augments vasodilation and vascular permeability, resulting in fluid extravasation. The gastrointestinal tract is one of several anatomically susceptible sites for bradykinin-mediated angioedema [7]. However, the reasons certain individuals develop angioedema in one anatomical location versus another remain incompletely understood.

Research on hereditary angioedema, another bradykinin-mediated condition, has proposed mechanistic models that may provide insight into ACEI-induced SBAE despite differing etiologies. Two primary models have been described: the contact activation model and the system-fluid phase model [8]. The contact activation model postulates dysregulated local control of the plasma contact system, leading to localized bradykinin generation and activation of nearby B2 receptors [8]. In contrast, the system-fluid phase model proposes a systemic elevation of bradykinin distributed via the circulation that then preferentially affects certain tissues. This framework may be more generalizable to ACEI-induced SBAE, where circulating bradykinin could disproportionately impact the gastrointestinal tract.

Genetic susceptibility may further influence anatomical localization. Ghouse et al. [5] identified a variant near the *BDKRB2* gene, which encodes the bradykinin B2 receptor. They propose that this variant may increase receptor expression or sensitivity to bradykinin, thereby increasing susceptibility to angioedema. Notably, colocalization of *BDKRB1*, encoding the bradykinin B1 receptor, was also observed. This distinction is mechanistically important. B2 receptors rapidly desensitize following bradykinin binding, limiting their ability to account for prolonged or localized swelling, such as that seen in SBAE [8]. In contrast, B1 receptors are inducible and upregulated on endothelial cells in response to inflammatory stimuli, including B2 receptor activation. Hofman et al. [8] suggest that B1 receptor induction may provide a more plausible explanation for sustained, tissue-specific edema. Further investigation is warranted to clarify the role of B1 receptor signaling in ACEI-induced SBAE.

There is a growing body of literature documenting ACEI-induced SBAE. The increasing number of case reports highlights the recognition and documentation of SBAE as an adverse effect of ACEI. Further, with increased utilization of "polypills" - a fixed-dose combination of multiple medications in a single pill, often including ACEIs and other medications, for cardiovascular disease prevention - some speculate there is an expected rise in angioedema prevalence [1].

In our literature review, other published case reports demonstrate overlap with our case, as well as some differences. For those impacted by ACEI-induced SBAE, the majority were middle-aged or older, and predominantly female [1]. Presenting symptoms typically included nonspecific abdominal symptoms, such as pain, nausea, vomiting, and diarrhea.

Imaging, particularly CT and MRI, is critical for diagnosis and often reveals bowel wall edema and ascites. A 2011 radiologic CT review of 20 patients with ACEI-induced SBAE found ascites in all patients, small-bowel wall thickening (mean, 1.3 cm), mild dilatation (mean, 2.9 cm), and straightening, with no signs of small-bowel obstruction [6]. Other case reports refer to these similar findings as a "target sign" on CT [9-13]. Other labs, which are pathognomonic for hereditary angioedema, such as C1 esterase inhibitor and complement C4, are not consistent with an ACEI-induced SBAE diagnosis. Further, lab findings such as WBC or ESR are not consistent across ACEI-induced SBAE presentations.

The time of onset varies significantly, with cases reported as early as 12 hours after initiation to as late as several years into therapy [14,15]. Management always included discontinuation of the ACEI, which led to symptom reduction within as few as 48-72 hours. In our case, years following discontinuation of ACEI, our patient remains without symptoms.

Our case highlights the challenge of diagnosing ACEI-induced SBAE, particularly in the context of patients with evolving medical histories and a wide differential diagnosis. As demonstrated, the patient underwent extensive evaluations over the years, including multiple imaging modalities, endoscopic procedures, and empiric treatment for suspected infectious etiologies or IBD. Not to mention significant economic losses and decreased quality of life.

Given the widespread use of ACEIs, clinicians must maintain a high index of suspicion for drug-induced angioedema, particularly when patients present with recurrent, unexplained abdominal pain. Additionally, this case underscores the importance of incorporating medication-induced etiologies into the differential diagnosis of long-term, unexplained symptoms. The healthcare team can play a pivotal role in systematically evaluating adverse drug reactions (ADRs).

Clinicians may opt to use assessment tools to review new and chronic patient symptoms for possible ADRs during routine visits. Methods for detecting and managing ADRs are generally beneficial to patients and have not been shown to cause harm, underscoring the need for further investment [16]. A systematic review of ADR detection in primary care offices suggests that a critical element of all ADR identification strategies involves comparing participants’ symptoms to a comprehensive list of potential ADRs. Combining these lists with prescriber consultations provided an effective approach to addressing associated challenges.

Additionally, many causality assessment tools have been developed to assess the likelihood that a patient’s ADRs are due to medication exposures, such as the World Health Organization Uppsala Monitoring Centre (WHO-UMC) system, Naranjo algorithm, Liverpool algorithm, updated Logistic method, and the Causality Documentation Tool (CausDoc). The WHO-UMC is generally considered favorable for its fair inter-rater agreement and consistency in hospital use, but its use in primary care settings needs further evaluation [17,18].

Further, physicians must remain vigilant in reporting all ADR findings to databases such as the FDA’s Adverse Event Reporting System (FAERS) public dashboard via the MedWatch platform. In the post-marketing phase, this system helps identify potential safety signs and support regulatory actions to ensure drug safety [19].

This case report is limited by its single-patient design, which restricts generalizability. Additionally, the seven-year retrospective timeline introduces potential recall bias, although medical records were reviewed to corroborate the clinical course. While symptom resolution following lisinopril discontinuation strongly supports causality, definitive confirmation without rechallenge is not feasible or ethical.

## Conclusions

In recent years, SBAE has been established as a possible adverse effect of ACEIs, which are commonly prescribed medications. This case illustrates the diagnostic challenges encountered over a seven-year period when evaluating patients presenting with nonspecific symptoms, gastrointestinal complaints, and overlapping pathologies. Resolution of symptoms following discontinuation of lisinopril emphasizes the need to consider drug-induced etiologies in patients with unexplained, persistent symptoms. The case further underscores the essential role clinicians can play in identifying and managing adverse drug reactions, particularly with frequently used medications such as ACEIs. Early recognition can reduce unnecessary hospitalizations and interventions and improve patient outcomes. Additionally, this case highlights the extensive diagnostic evaluations, increased healthcare burden, and patient fatigue that may result from delayed diagnosis.

## Appendices

Patient perspective

Note received February 2025; text is in brackets where details were anonymized or details added.

In April 2015, I started feeling nausea and then abdominal pain that radiated from my mid-abdomen slowly to my back and forth. It was intermittent pain similar to labor pain. It would start as mid pain and radiate to excruciating pain. I commenced vomiting and diarrhea at the same time. Even after I vomited all the food, I would then vomit bile. I was taken to the ER, where they administered IV fluids, morphine for the pain, and anti-nausea medication. I was dehydrated, and my white cell count was elevated. They ordered a CT scan that indicated I had inflammation, and it was [*clinically*] diagnosed as gastroenteritis. They prescribed antibiotics and anti-nausea medications, along with pain medication as needed. I was recommended to consume clear liquids and to follow up with my primary doctor. I followed the doctor's instructions to the letter.

My primary doctor referred me to my first gastroenterologist, who performed an endoscopy and colonoscopy. I mentioned that it may be my gallbladder, as all females in my family had to have theirs removed. He stated I had a hiatal hernia and acid reflux, and my gallbladder looked fine. He ordered a test on the bladder functionality and said it was okay. He prescribed pantoprazole and recommended not to drink acidic drinks such as juice or alcohol, which I rarely drink anyway. I stopped drinking any kind of juice or alcohol. However, I continued to experience episodes every two months of pain, vomiting, and diarrhea, which dehydrated me and required me to be taken to the ER. Inflammation in the small bowel was always present. They treated me the same way as the other times.

In July 2017, I was hospitalized due to extreme small bowel inflammation. An MRI was done, along with another colonoscopy and endoscopy. The same medications and treatment were administered. I followed up with the hospital attending gastroenterologist, a graduate from [*a school*]-my second gastroenterologist-and I thought they would be able to find out why I kept experiencing this pain. I also mentioned my family's female history of gallbladder removals. He too said everything was good. They began sending me for testing for Crohn's disease since my nephew has it, but it was negative. They sent me for a swallowing test, which was unpleasant. They ordered a pill endoscopy/colonoscopy, and nothing was found. He sent me to a Crohn's specialist who, on my initial visit, told me he did not think I had Crohn's because the inflammation was never in the same spot, but he would check. I asked him to please check my gallbladder and the reasons why, and he said that he would check. After the procedure on October 27, 2017, he said he was right-there was no sign of Crohn's; however, he recommended that the gallbladder be removed. I was so happy that finally the cause would be addressed. It took the gastroenterologist two weeks before referring me to a surgeon. I had to have an appointment first with the surgeon on November 17, 2017. I went through another episode of pain and wanted to avoid returning to the ER, so I contacted my primary doctor's office, which had recently hired a specialist who became my third gastroenterologist to their team. She kindly agreed to see me, but due to the level of pain I was experiencing, I reached out to the surgeon's office and requested an immediate appointment. After seeing the surgeon, he directed me straight to the ER with instructions for admission and surgery scheduled for the following morning. I waited in the ER for six hours because they were at capacity and could not admit me, despite the doctor's orders. That evening, the surgeon visited the ER and informed me that he had reviewed my medical records and noted the inflammation was severe. He didn’t believe it was related to my gallbladder and suggested an exploratory procedure. I agreed, stating that as long as he removed the gallbladder, he could proceed with the exploration. The morning before surgery, I reiterated my request for the gallbladder to be removed. He explained that if the gallbladder appeared healthy, he would not be able to do so. On November 17, 2017, after the surgery, he informed me that it was the worst gallbladder he had ever extracted. I felt fantastic and was convinced this would put an end to my pain episodes. However, I continued to suffer from those pains and had to visit the ER every three months. Now, I would no longer need to go to the ER.

In February 2018, the pain returned. I went back to the ER again and followed up with my gastroenterologist. She sent me for allergy tests, blood work of all kinds, and a nuclear MRI; however, all results were good. She left the practice I believe was 2019, and I had to find my fourth gastroenterologist. She was as good as my last one, very compassionate and also curious as to why they couldn’t find the cause. She ran all the same tests as well. I had to be hospitalized again in 2021 due to another episode, plus extreme weakness and low potassium; it was during the pandemic too. During my stay, she ordered another balloon endoscopy because of my family medical history. It was negative. She ran more blood work and a fecal matter test. I tested gluten intolerant. My calcium was low, which was affecting my bones, and she referred me to an endocrinologist and a dietitian to help guide me on what to eat. My fourth gastroenterologist moved back to [*home*], and I stayed with the [*the same gastroenterology group*] and my fifth gastroenterologist, who was baffled as to why I kept experiencing the pain episodes. He sent me for another balloon endoscopy in October 2022, which was negative for Crohn's, then another pill endoscopy/colonoscopy, which were all negative. Then, in my April 2023 appointment, he told me he wanted to speak to my cardiologist because he did some research on lisinopril and found that there are rare cases where patients on lisinopril, similar to me, have experienced edema of the small bowel. He recommended that my cardiologist change my medication to see if it would relieve the abdominal episodes I had been experiencing.

As of April 2023, I stopped taking lisinopril, and I have not had one abdominal episode. I feel great and am so thankful that my fifth gastroenterologist took the time to research the medications I was on to see if there were any side effects that could cause what I was experiencing.

The past seven years have been incredibly challenging for me, as I was always the healthy and strong member of my family. My family was deeply concerned and feared the worst, suspecting it could be cancer or another incurable condition. I faced significant medical expenses and felt utterly powerless, especially after consulting numerous specialists who offered no explanations. I am grateful that the fifth doctor finally identified the issue. If my experience can assist any physician in researching medications, I urge patients to inquire or insist that doctors investigate the rare side effects of medications that might resemble their symptoms.

I would like to express my gratitude to [*my primary care team*], for your interest in my experience as a case study on Lisinopril.
